# Genome sequencing accuracy by RCA-seq versus long PCR template cloning and sequencing in identification of human papillomavirus type 58

**DOI:** 10.1186/2045-3701-4-5

**Published:** 2014-01-13

**Authors:** Xiaohong Wang, Yang Li, Ting Ni, Xing Xie, Jun Zhu, Zhi-Ming Zheng

**Affiliations:** 1 Tumor Virus RNA Biology Section, Gene Regulation and Chromosome Biology Laboratory, Center for Cancer Research, National Cancer Institute, National Institutes of Health, Bethesda, MD, USA; 2 Department of Gynecologic Oncology, Women's Hospital, School of Medicine, Zhejiang University, Hangzhou, China; 3 DNA Sequencing and Computational Biology Core, National Heart Lung and Blood Institute, National Institutes of Health, Bethesda, MD, USA; 4 State Key Laboratory of Genetics Engineering and Ministry of Education Key Laboratory of Contemporary Anthropology, School of Life Sciences, Fudan University, Shanghai, China

**Keywords:** Human papillomaviruses, HPV58, Cervical cancer, Single nucleotide polymorphism, Genotyping, Genome variations, Rolling circle amplification, DNA deep sequencing

## Abstract

**Background:**

Genome variations in human papillomaviruses (HPVs) are common and have been widely investigated in the past two decades. HPV genotyping depends on the finding of the viral genome variations in the L1 ORF. Other parts of the viral genome variations have also been implicated as a possible genetic factor in viral pathogenesis and/or oncogenicity.

**Results:**

In this study, the HPV58 genome in cervical lesions was completely sequenced both by rolling-circle amplification of total cell DNA and deep sequencing (RCA-seq) and by long PCR template cloning and sequencing. By comparison of three HPV58 genome sequences decoded from three clinical samples to reference HPV-58, we demonstrated that RCA-seq is much more accurate than long-PCR template cloning and sequencing in decoding HPV58 genome. Three HPV58 genomes decoded by RCA-seq displayed a total of 52 nucleotide substitutions from reference HPV58, which could be verified by long PCR template cloning and sequencing. However, the long PCR template cloning and sequencing led to additional nucleotide substitutions, insertions, and deletions from an authentic HPV58 genome in a clinical sample, which vary from one cloned sequence to another. Because the inherited error-prone nature of *Tgo* DNA polymerase used in preparation of the long PCR templates of HPV58 genome from the clinical samples, the measurable error rate in incorporation of nucleotide into an elongating DNA template was about 0.149% ±0.038% in our studies.

**Conclusions:**

Since PCR template cloning and sequencing is widely used in identification of single nucleotide polymorphism (SNP), our data indicate that a serious caution should be taken in finding of true SNPs in various genetic studies.

## Introduction

Human papillomaviruses (HPVs) are a group of more than 200 genotypes of small DNA tumor viruses ( http://pave.niaid.nih.gov/#home) and can be grouped clinically as high-risk (oncogenic) types, which are frequently associated with invasive cervical cancer, and low-risk (non-oncogenic) types, which are found mainly in genital warts. To date, fifteen HPV types has been classified as high-risk HPVs, including HPV16, 18, 31, 33, 35, 39, 45, 51, 52, 56, 58, 59, 68, 73, and 82 [1,2]. Among the high-risk HPVs, HPV16 and HPV18 are the principal causes of cervical cancer, with a combined, worldwide contribution to ~70% of invasive cervical cancer [3,4]. HPV58 has been found to be more prevalent than HPV18 in cervical intraepithelial neoplasia (CIN) lesions and appears almost equal frequency as HPV18 in cervical cancers in Zhejiang province, China [5] and other Asian countries [6,7]. We recently characterized HPV58 genome variations and RNA expression in women with cervical lesions [8].

HPV genome in size of ~7.9 kb encodes eight open reading frames (ORFs, E6, E7, E1, E2, E4, E5, L2 and L1) from one strand of its double stranded, circular genome in one direction and a long control region (LCR) between L1 and E6 [9]. The classification of papillomaviruses by genotyping depends on the most conserved L1 ORF. A new type of papillomavirus is granted when its DNA sequence of L1 ORF differs by more than 10% from the closest known HPV type. A subtype indicates the difference between 2% and 10% and a variant represents less than 2% difference [10-12]. Nucleotide variations in the LCR region are often used to describe intratype diversity or variant lineage [12-15]. Despite that HPV genome is viewed as a stable genome, its genome sequence variations in a given genotype appear different from one laboratory to another and from one geological region to another [14-22]. The reported variations have been found not only in the L1 ORF and the LCR region, but also in other parts of the viral genome and were identified mostly, if not all, by PCR amplicon sequencing. Although it is always questionable whether an authentic variation (s) does exist in the reported HPV genotype because of the use of error-prone *Taq* polymerase in PCR amplification, many attempts have been made to correlate such genome variations to HPV pathogenesis and viral carcinogenicity [23-26]. Thus, a more reliable method is urgently needed to study HPV genome variation and its possible biological role in HPV infection. Recently, next-generation sequencing (NGS) techniques are emerging as a promising application for HPV genotyping and identification of rare single nucleotide polymorphism (SNP) [8,27-30].

In this report, we analyzed and compared three HPV58 genome sequences from three cervical lesion samples decoded by an RCA-seq (rolling-circle amplification and deep sequencing) [8,31], a technique that does not require prior knowledge of the underlying genome, and by long PCR template cloning and Sanger sequencing. We demonstrated that RCA-seq is more reliable method in identification of an authentic nucleotide variation in an episomal viral genome.

## Results

**Figure 1:**
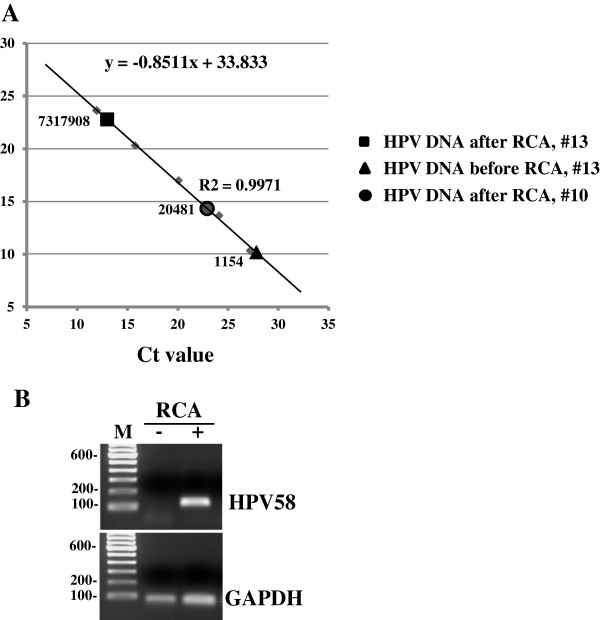
**Enrichment of HPV genomic DNA by RCA from cervical samples. (A)** HPV58 genome copy numbers before and after RCA enrichment. Real-time PCR (qPCR) was performed with an HPV58-specific primer pair on ~100 pg of sample DNA (sample 10 and sample 13) either before or after RCA enrichment. A 10-fold serial dilution, starting from 100 pg (~1.3 x 10^7^ copies) of the plasmid pXW59-1 which contains an HPV58 DNA fragment from nt 6906 to 3695 was amplified using the same primer set by qPCR to create a standard curve. The threshold cycle (Ct) values of qPCR data from 2 repeats were calculated for copy number analysis. GAPDH was used as an internal control. **(B)** HPV58 DNA in the sample 10 was under detection level before RCA, but became detectable by agarose gel electrophoresis after enrichment by RCA to 20481 copies as quantified by qPCR.

To analyze single nucleotide polymorphism (SNP) in HPV genome, RCA was applied to enrich copy number of an episomal HPV58 genome from each of three cervical lesion samples. To quantify HPV58 and host GAPDH copy numbers from the same sample before and after RCA, a quantitative real-time PCR (qPCR) was performed on ~100 pg of sample DNA (samples 10 and 13). The threshold cycle (Ct) values from 2 repeats were calculated for copy number analysis. Human GAPDH DNA in linear form served as an internal control. As expected, we found that RCA enriched HPV58 genome copy number in the sample 13 from 1154 copies to 7317908 copies (more than 6300-fold enrichment) while displaying no enrichment activity to host GAPDH linear DNA. The Ct values for GAPDH DNA were 23.42 before RCA enrichment and 22.76 after RCA enrichment in the sample 13. A similar result was observed in the sample 10, in which HPV58 genome copy was under detection level before RCA, but could be enriched by RCA to 20481 copies quantified by qPCR (Figure 1A) and became detectable by agarose gel electrophoresis (Figure 1B), with only a little enrichment of host GAPDH linear DNA.

To have the RCA products being deep-sequenced using an Illumina HiSeq-2000 platform, we subsequently debranched the RCA products and prepared a paired-end library from each RCA sample [8,31]. More than 104 millions of paired-end reads from the sample 9, 161 millions from the sample 10, and 120 millions from the sample 13 were obtained using the RCA-seq. Among those reads, 3453 in the sample 9, 13249 in the sample 10, and 1.4 million in the sample 13 could be mapped to the reference HPV58 genome (GI: 222386) [32], giving a complete coverage of a full-length HPV58 genome [8]. Detailed analyses of the nucleotide sequence at each position against the reference HPV58 genome [32] showed that the HPV 58 genomes in three clinical samples contain nucleotide substitutions being verifiable either in other samples or in the same sample by the second RCA-seq [8].

We subsquently cloned the full-length, episomal HPV58 genome from each clinical sample and compared each genome sequence obtained by primer-walking Sanger sequencing to the genome sequence obtained by the RCA-seq. To do so, the same RCA product from each clinical sample prepared for RCA-seq, without S1 digestion and DNA shearing, was linearized by AgeI digestion at nt 62 position or by DraII digestion at nt 4536 position of HPV58 genome. Subsequently, two large fragments, a ~3.5-kb fragment from nt 3506 to 7036 of HPV58 genome linearized from AgeI-digested RCA product and a ~4.6-kb fragment from nt 6905 to 3694 of HPV58 genome linearized from DraII-digested RCA product from each clinical sample, were amplified separately by high-fidelity thermostable *Tgo* DNA polymerase with proofreading activity and inserted separately into a pCR-XL-TOPO vector. Two clones for each fragment and four clones for each RCA product of the individual clinical sample were obtained (Table 1). All plasmid clones were then sequenced by conventional primer-walking Sanger sequencing on both strands and the full-length viral genome sequence was aligned against the reference HPV58 genome and its corresponding HPV58 genome as determined by RCA-seq.

**Table 1:** The list of plasmids constructed and used in the study

**Plasmid**	**Characteristics**
pXHW54	RCA product from HPV58 CNZJ-3 (sample 9) was amplifiedwith Pr3506 (oYL35) and Pr7036 (oXHW262), gel purified andcloned into pCR-XL-TOPO vector. Insertion was verified bysequencing.
pXHW55	RCA product from HPV58 CNZJ-2 (sample 10) was amplifiedwith Pr3506 (oYL35) and Pr7036 (oXHW262), gel purified andcloned into pCR-XL-TOPO vector. Insertion was verified by sequencing.
pXHW56	RCA product from HPV58 CNZJ-1 (sample 13) was amplified with Pr3506 (oYL35) and Pr7036 (oXHW262), gel purified and cloned into pCR-XL-TOPO vector. Insertion was verified by sequencing.
pXHW57	RCA product from HPV58 CNZJ-3 (sample 9) was amplified with Pr6906 (oXHW263) and Pr3694 (oXHW264), gel purified and cloned into pCR-XL-TOPO vector. Insertion was verified by sequencing.
pXHW58	RCA product from HPV58 CNZJ-2 (sample 10) was amplified with Pr6906 (oXHW263) and Pr3694 (oXHW264), gel purified and cloned into pCR-XL-TOPO vector. Insertion was verified by sequencing.
pXHW59	RCA product from HPV58 CNZJ-1 (sample 13) was amplified with Pr6906 (oXHW263) and Pr3694 (oXHW264), gel purified and cloned into pCR-XL-TOPO vector. Insertion was verified by sequencing.

Two bacterial colonies were picked up for each plasmid preparation and the obtained plasmid preps were named as a plasmid-1 or -2 (such as pXHW54-1 or -2 described in the text).

**Figure 2:**
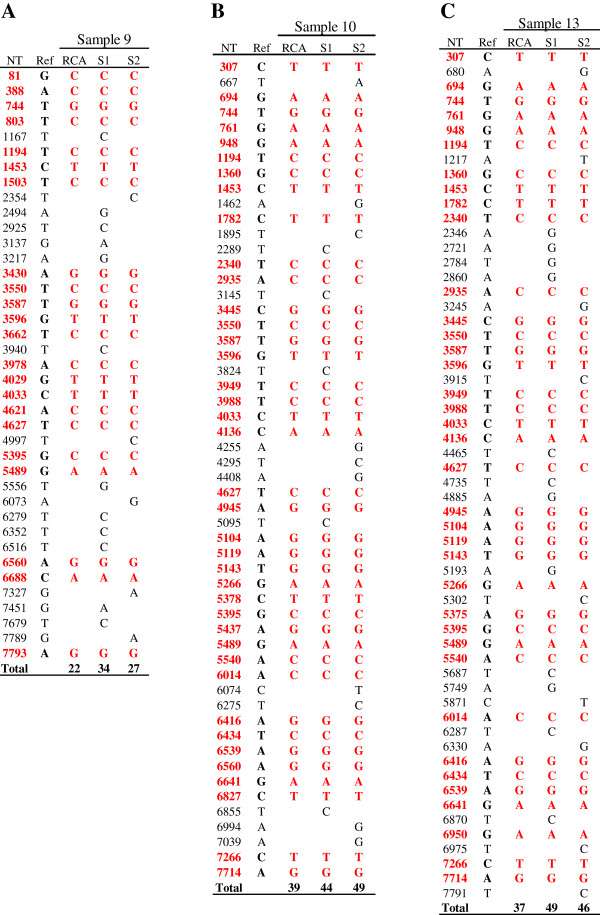
**Nucleotide substitutions identified in HPV58 isolates to the reference HPV58 **[32]** by RCA-seq and by cloning sequencing.** RCA-seq results were compared to the sequencing results of two individual bacterial clones of each plasmid for samples 9 **(A)**, 10 **(B)**, and 13 **(C)**. S1 and S2 denotes Sanger sequence #1 (clone #1) and #2 (clone #2), respectively. Common nucleotide substitutions at positions in the reference HPV58 genome seen from RCA-seq to cloning sequencing were colored in red. The nucleotide substitutions identified only by cloning sequencing were shown in black.

**Figure 3:**
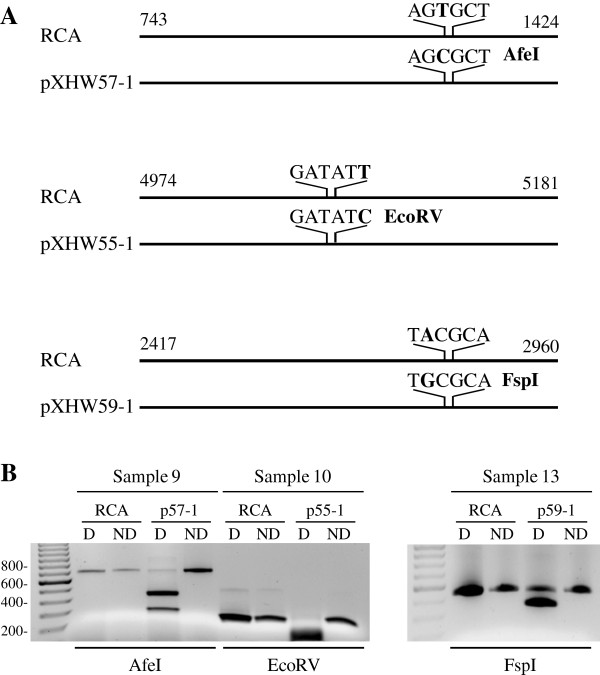
**Restriction enzyme digestion distinguishes HPV58 amplicons from RCA products to their corresponding plasmids. (A)** Diagrams of HPV58 amplicons from RCA samples and their corresponding plasmid clones with a new restriction enzyme cutting site. **(B)** Restriction enzyme digestion of RCA products and their corresponding plasmid clones from each clinical sample. RCA products and pXHW57-1 from the sample 9 were amplified using a primer pair of Pr743 and Pr1424 and digested with AfeI. RCA products and pXHW55-1 from the sample 10 were amplified using a primer pair of Pr4974 and Pr5181 and digested with EcoRV. RCA products and pXHW59-1 from the sample 13 were amplified using a primer pair of Pr2417 and Pr2960 and digested with FspI. The digested products were resolved in 1.5% agarose gel. D: digested; ND: not digested.

As shown in Figure 2, 22 nucleotide substitutions in the sample 9-derived HPV58 (CNZJ-3, GenBank accession number KC860270), 39 in the sample 10-derived HPV58 (CNZJ-2, GenBank accession number KC860271), and 37 in the sample 13-derived HPV58 (CNZJ-1, GenBank accession number KC860269) found in RCA-seq [8] were all verified in the HPV58 plasmid clones derived from individual clinical samples, but the long PCR template cloning and sequencing created additional nucleotide variations to its corresponding viral genome sequence determined by RCA-seq. The sample 9-derived plasmid sequence-1 (seq-1) decoded from pXHW54-1 plus pXHW57-1 contained additional twelve nucleotide substitutions and four nucleotide insertions and its seq-2 from pXHW54-2 plus pXHW57-2 contains five separate nucleotide substitutions, two separate nucleotide insertions, and one nucleotide deletion (Figure 2 and Table 2). For the sample 10-derived plasmid seq-1 decoded from pXHW55-1 plus pXHW58-1 and the seq-2 from pXHW55-2 plus pXHW58-2, the additional nucleotide variations include five substitutions, two insertions, and one deletion in its seq-1 and ten separate substitutions in its seq-2 (Figure 2 and Table 2). Similarly, the viral genome sequence derived from the sample 13 displayed additional twelve nucleotide substitutions and two nucleotide insertions in its seq-1 decoded from pXHW56-1 plus pXHW59-1 and nine separate substitutions and one separate insertion in its seq-2 decoded from pXHW56-2 plus pXHW59-2 (Figure 2 and Table 2). Further analyses of the additional variations generated by the long PCR template cloning and sequencing showed that some of them could create or inactivate a restriction enzyme cutting site as summarized in Table 3 and could be therefore verified by the restriction enzyme digestion. As shown in Figure 3, AfeI, EcoRV, or FspI could digest the PCR products, respectively, from pXHW57-1, pXHW55-1, or pXHW59-1, but not the PCR products from their corresponding RCA preps derived from the same clinical sample, indicating that these nucleotide variations do not exist in the authentic HPV58 genome, rather they were most likely introduced by the long PCR template cloning and sequencing.

**Table 2:** Nucleotide insertion and deletion created by long-PCR amplification in HPV58 isolates

	**Insertion**						**Deletion**					
	**Sample 9**		**Sample 10**		**Sample 13**		**Sample 9**		**Sample 10**		**Sample 13**	
**at nt**	**S1**	**S2**	**S1**	**S2**	**S1**	**S2**	**S1**	**S2**	**S1**	**S2**	**S1**	**S2**
2153	A											
2253			T									
2265	A		A									
3081									T			
3083	AA											
3083								A				
3767		TT										
4460					CC							
5632						T						
**Total**	**4**	**2**	**2**	**0**	**2**	**1**	**0**	**1**	**1**	**0**	**0**	**0**

Summarized in this table are nucleotide insertions/deletions introduced by long PCR amplification from the two individual clones in each of three HPV58 isolates. S1 and S2 denotes Sanger sequence #1 (clone #1) and #2 (clone #2), respectively. Numbers are nucleotide positions in the reference HPV58 genome at where the insertion or deletion was identified in each HPV58 isolate.

**Table 3:** Nucleotide substitutions by long-PCR amplification in HPV58 isolates create or inactivate restriction enzyme digestion sites

		**Sample number**		
**Enzyme**	nt position*	**9**	**10**	**13**
AfeI	1167	+	-	-
FspI	2860	-	-	+
DraIII (inactive)	2925	+	-	-
FspI	3940	+	-	-
EcoRV	5095	-	+	-
BstXI	5556	+	-	-
MscI (inactive)	6352	+	-	-
EaeI	6352	+	-	-

Summarized in this table are distinguishable restriction enzyme cutting sites due to nucleotide substitutions introduced by long PCR amplification in three HPV58 isolates. See other details from Figure 3 legends. *The numbers are nucleotide positions with the substitutions to the reference HPV58 genome.

Notably, these additional nucleotide variations from the long PCR template cloning and sequencing were random and not duplicable each other between two cloned viral genome sequences derived from the same clinical samples. In addition, we found that the insertions or deletions identified from the cloned HPV58 genome fragment always happened at a run of multiple A, T, or C. Together, a total of additional 70 nucleotide variations were identified from six cloned HPV58 genome sequences which differ from the RCA-seq-defined HPV58 genome sequences. We concluded that these variations were derived from long PCR template amplification with a less efficient proof-reading *Tgo* DNA polymerase. By calculation, the *Tgo* DNA polymerase exhibited an error rate of ~0.149% ±0.038% in our long PCR template preparation per HPV58 genome in size of 7824 nts.

## Discussion

Rolling-circle amplification (RCA) has been developed as a powerful tool to amplify a whole genome in an episomal form for microbial genome organization and phylogenetic analyses [33]. In this study, HPV58 genome in cervical lesions was decoded, respectively, by RCA-seq and by long PCR template cloning and sequencing. We demonstrated that the RCA-seq is better than the long PCR template cloning and sequencing in providing more accurate HPV58 genome sequence. This conclusion was drawn by comparing the same genome sequence decoded by the two techniques. All nucleotide variations to the reference HPV58 genome identified from each clinical sample by RCA-seq could be verified by the long PCR template cloning and sequencing, but not vice versa. In addition, the long PCR template cloning and sequencing technique creates additional sequence variations, which were random and not verifiable from one cloned sequence to another derived from the same sample.

Although the viral DNA from each clinical sample was enriched by RCA using phi29 DNA polymerase [34,35] which features only a minimal error rate of 1 in 10^6^-10^7^[36] and the reported sequencing accuracy for a NGS with Illumina HiSeq platform is 98% [37], we found that RCA-seq in this report displayed a more reliable approach, by calling consensus bases, in decoding the entire HPV58 genome in size of 7824 nts. This could be achieved by using a combination of both high fidelity phi29 DNA polymerase at the experimental side and appropriate sequencing data analysis at the computational side. First, we defined a position as identical if more than 95% of the bases obtained were identical to those of the reference genome. Second, nucleotide substitutions were identified if the majority of bases in the reads differ from the reference genome with read depth of 5 or more. Third, nucleotide positions with read depth less than 5 were treated as ambiguous sites since there is insufficient depth to make a high confidence call [8].

We determined that additional nucleotide variations observed in our long PCR template cloning and sequencing were derived from the proofreading error of *Tgo* DNA polymerase used in our long PCR template preparation. Although the enriched DNA chimeras in the RCA reaction which might carry a misincorporated base from phi29 DNA polymerase amplification were served, after debranching by AgeI- or DraII-specific digestion of HPV58 genome, for subsequent long PCR template preparation and cloning, this carry-over possibility appears to be unlikely because the phi29 DNA polymerase features only a minimal error rate of 1 in 10^6^-10^7^[36] in contrast to ~3 in 10^4^ for PCR with *Taq* DNA polymerase [38]. Other studies indicate that the majority of DNA sequence changes introduced during PCR are polymerase-mediated and PCR accumulates about one mutation per 400 bases after 30 cycles [38,39]. The PCR-induced transitions are the major source of error in other NGS studies [40,41]. The reported *Tgo* DNA polymerase from the Roche product manual has an overall error frequency of ~0.2% in nucleotide misincorporation during PCR amplification, very close to what we found in our study.

In summary, a more accurate RCA-seq superior to PCR cloning and sequencing has been developed in our lab for HPV genotyping and determination of SNPs in clinical HPV variants. Our protocol provides much higher numbers of specific HPV reads [8] over other high-throughput NGS platforms/protocols applied to HPV genotyping [27-30]. Since PCR sequencing and PCR template cloning and sequencing are widely used in various epidemiology studies to identify natural variants of an HPV type, caution should be taken in evaluation of any new SNPs found by these assays as PCR amplification might introduce nucleotide misincorporation into the studying genotype (s).

## Materials and methods

### Sample DNA preparation

Three HPV58- positive cervical CIN2/3 tissues were collected from Women's Hospital, School of Medicine, Zhejiang University. The study was approved by the Institutional Review Board for clinical research of this hospital. Informed consent was obtained from each participant prior the study. DNA was isolated from each sample by using TRIzol Reagent (Invitrogen, Carlsbad, CA) according to the manufacturer's instructions.

### RCA enrichment, RCA chimeras debranching, paired-end library preparation and deep sequencing

Rolling circle amplification (RCA) based on phi29 DNA polymerase used to enrich the HPV58 genome and RCA chimeras debranching, paired-end library preparation and deep sequencing with an Illumina HiSeq-2000 platform have described in our other publications [8,31].

### Long PCR template preparation, cloning, and sequencing

RCA products prepared separately from three clinical samples (sample 9, 10 and 13) without S1 digestion and DNA shearing were selectively linearized by AgeI or DraII digestion and then amplified with a primer pair Pr3506 (5′-GACAGTAGACCACGAGGA-3′, forward) and Pr7036 (5′-TACTCAGGATC/CGTCCCAAAGGAAACTGATC-3′, backward) for AgeI-digested RCA products, and with a primer pair Pr6906 (5′-TACATCGAATT/CTCCCAGGCTATTACTTGC-3′, forward) and Pr3694 (5′-CCAATGCCATGTGGATGAC-3′, backward) for DraII-digested RCA products, respectively, using the Expand Long Template PCR System (Roche, Cat No. 11681834001). In brief, 2 μl of digested RCA products were used for amplification in a 50-μl reaction together with 38.75 μl DEPC-treated water, 2.5 μl dNTP mix (10 mM each)), 0.5 μl of each forward and reverse primers (20 μM), 5 μL of 10x PCR buffer II with MgCl2, and 0.75 μl of the expand long template enzyme mix which contains high-fidelity thermostable *Tgo* DNA polymerase with proofreading activity. Individual long PCR product was gel-purified and cloned into pCR-XL-TOPO vector according to the manufacturer’s instruction (Invitrogen, Cat No K4700-10). In brief, the PCR products generated from AgeI-digested RCA products using primer pairs Pr3506 (forward) and Pr7036 (backward) and the PCR products generated from DraII-digested RCA products using primer pairs Pr6906 (forward) and Pr3694 (backward) were precipitated, purified by agarose gel electrophoresis using crystal violet and then isolated using S.N.A.P Gel Purification Kit (Invitrogen). Purified long PCR products were cloned into a pCR-XL-TOPO vector. Two clones from each plasmid (Table 1) were prepared and sequenced from two different directions. Sequencing results of each clone were aligned against the HPV58 reference genome to identify nucleotide substitutions, insertions, or deletions.

### Restriction enzyme digestion

RCA product and plasmid pXHW57-1 from the clinical sample 9 were amplified with primer pair Pr743 (5′-CGTGTTGTTACACTTGTGAC-3′) and Pr1424 (5′-GTATTACAACTGTCTACATCCG-3′), and then digested with AfeI at 37°C overnight. RCA product and pXHW55-1 from the sample 10 were amplified with primer pair Pr4974 (5′-CATCTCCTCATAGACTTGTAAC-3′) and Pr5181 (5′-CGAGTACGAAGTGTAGCCT-3′), and then digested with EcoRV at 37°C overnight. RCA product and pXHW59-1 from the sample 13 were amplified with primer pair Pr2417 (5′-GTATGATAGATGATGTAACAGC-3′) and Pr2960 (5′-ACGCTTTAGTCTTTGATGCTA-3′), and then digested with FspI at 37°C overnight. Non-digested products without enzymes were used as controls.

### Sequencing data analyses

The trimmed reads were aligned to human (hg19 assembly) from UCSC genome browser and HPV58 reference genome (GenBank accession number D90400 or GI:222386) from PaVe database simultaneously using the Burrows-Wheeler Alignment tool (BWA) with default setting [42]. The output alignment files in SAM format were further processed using SAMtools [43]. The genome coverage files (WIGGLE or BAM files) were loaded onto the Integrative Genomics Viewer (IGV, http://www.broadinstitute.org/igv) to visualize sequence alignments, genomic annotations and substitutions [44] with defaulted settings. The cloning sequences were aligned to HPV58 reference genome using NCBI Blast tool.

### Quantitative real-time PCR

Quantitative real-time PCR, with an HPV58-specific primer pair Pr 743 (5′-CGTGTTGTTACACTTGTGAC-3′) and Pr 854 (5′-CTAGGGCACACAATGGTACA-3′) using Power SYBR Green PCR Master Mix (Invitrogen) for high sensitivity and reproducibility, was performed on ~100 pg of samples DNA (sample #10 and #13) before and after RCA enrichment, of which the RCA products from clinical samples #10 and #13 without S1 digestion and DNA shearing were selectively linearized by AgeI or DraII digestion. A 10-fold serial dilution starting from 100 pg (~1.3 x 10^7^ copies) of the plasmid pXW59 which contains an HPV58 DNA fragment from nt 6906 to 3695 was amplified using the same primer set by qPCR to create a standard curve. The threshold cycle (Ct) values of qPCR data from 2 repeats were calculated for copy number analysis. A primer pair optimized for routine SYBR Green real-time PCR assays which specifically amplifies a genomic region containing human GAPDH promoter was purchased from Diagenode (Denville, NJ) and was used an internal control.

## Competing interests

The authors declare that they have no competing interests.

## Authors’ contributions

All authors conceived, designed and analyzed the study. XW, YL and TN performed experiments, with YL and TN on RCA-seq and XW on long PCR template cloning and sequencing and all other assays. YL and XX collected clinical samples and performed HPV screening. XW, YL and TN wrote the draft manuscript. ZMZ, JZ and XX finalized the manuscript. All authors read and approve the final manuscript.
